# Key Considerations for an Economic and Legal Framework Facilitating Medical Travel

**DOI:** 10.3389/fpubh.2016.00047

**Published:** 2016-03-31

**Authors:** Saba Hinrichs-Krapels, Sarah Bussmann, Christopher Dobyns, Ondřej Kácha, Nora Ratzmann, Julie Holm Thorvaldsen, Kai Ruggeri

**Affiliations:** ^1^The Policy Institute, King’s College London, London, UK; ^2^Department of Psychology, University of Cambridge, Cambridge, UK; ^3^Department of Politics and International Relations, University of Oxford, Oxford, UK; ^4^Department of Psychology, Faculty of Arts, Masaryk University, Brno, Czech Republic; ^5^Department of Social Policy, London School of Economics, London, UK; ^6^Department of Psychology, Norwegian University of Science and Technology, Trondheim, Norway; ^7^Policy Research Group, Department of Psychology, University of Cambridge, Cambridge, UK; ^8^Engineering Design Centre, Department of Engineering, University of Cambridge, Cambridge, UK

**Keywords:** medical travel, economic and legal framework, public policy, medical travel process, adverse effects

## Abstract

Medical travel has the capacity to counter increasing costs of health care by creating new markets and increased revenue for health services, potentially benefiting local populations, economies, and health-care systems. This paper is part of a broad, comprehensive project aimed at developing a global health access policy (GHAP). It presents key issues to consider in terms of ensuring economic viability, sustainability, and limiting risk to the many stakeholders involved in the rapidly expanding industry of medical travel. The noted economic and legal barriers to medical travel are based on a synthesis of themes found in an extensive review of the available literature. Economic considerations, when setting up a GHAP, include a dynamic approach to pricing that is fair to the local population. Legal considerations include the implementation of international quality standards and the protection of the rights of those traveling as well as those of local populations in recipient countries. By taking into account these opportunities, the GHAP will more adequately address existing gaps in the economic and legal regulation of medical travel.

## Introduction

In contrast to medical tourism, which broadly describes the movement of individuals for non-urgent treatments (e.g., cosmetic surgeries) or recreational and leisure purposes (1), the definition of medical travel explicitly refers to patients crossing national borders with the purpose of receiving treatment that has been determined as essential to maintaining quality of life by a health professional, but may not need to be performed urgently (2). The work reported in this paper is part of a broad, comprehensive project aimed at developing a global health access policy (GHAP) to facilitate safety, efficiency, and equal access in medical travel, as outlined by the Organisation for Economic Co-operation and Development (OECD) in the context of medical tourism (1). This work establishes the economic and legal perspective of a GHAP, identifying the key issues to consider when setting up such a policy to ensure economic viability and sustainability, as well as limiting risk to the stakeholders involved.

An increase in medical travel might result in a broad spectrum of consequences, e.g., (a) a wide-ranging impact on health-care systems (3, 4), (b) access of inbound countries (countries which receive medical travelers) to a larger market and additional revenue streams (5), and (c) easier access of patients in outbound countries (countries which send medical travelers) to medical care, reduced costs, and better standard of care (1). However, the uncontrolled movement of patients carries significant risks for both patients and health-care systems with respect to quality standards, dissemination of diseases, financial implications of adverse events, and unregulated developments on the health-care market (6, 7). Accordingly, the costs and benefits of medical travel as well as associated ethical and legal concerns call for a systematic evaluation to ensure the economic viability and sustainability of medical travel.

Several agreements that focus on facilitating medical travel already exist in certain regions, including those between the EU member states (8), within North America (NAFTA), and between OECD members such as the United States and Korea (1). For example, the EU’s Patients’ Rights Directive supports policies within the EU that ensures that patients making use of medical services abroad are entitled to receive financial reimbursements and follow-up care in their home country (8). However, this is still a directive and not regulation, and therefore, the extent to which financial support is given can differ for each member state. Furthermore, the existing frameworks often fail to consider the much wider needs of patients traveling for care, such as protection and indemnification, psychosocial support during rehabilitation and long-term liability, as well as ensuring access and equity (7, 9). Globally, therefore, medical travel remains a widely unregulated extension of health care (10).

As such, GHAP seeks to address existing gaps in the regulation of medical travel. There is a need for more evidence-based, patient-centered, and population-centered frameworks ensuring positive outcomes for all stakeholders, accounting for potential areas of increased risk and improving sustainability and equity. In the following section, we focus on the economic and legal considerations required for the development of such frameworks.

## Consequences and Concerns of Medical Travel

This paper focuses on the economic and legal consequences of medical travel (specified earlier as essential and non-cosmetic care), from the moment a patient starts to consider the option of medical travel, through receiving treatment and posttreatment care, to returning to the outbound country. Our aim was twofold: first, to broadly identify from existing literature the economic and legal risks of medical travel, given its aforementioned global increase, and second, to synthesize these risks and provide key considerations on how to establish a legal and economic framework for medical travel. We note that we did not intend to provide a full breakdown of the costs and benefits of medical travel as this was out of scope of our project and not justified by our broad literature search. A non-systematic review of scientific and gray literature was conducted. Articles were considered if they were found in any of the six databases under the terms “medical tourism” or “medical travel” since the year 2000, with no language restrictions. The databases used were PubMed, EconLit, Google Scholar, the World Bank research database, Europe PubMed Central, and EMBASE.

We first identified primary themes to be reviewed through common arguments used in high-quality sources as well as through common topics in low-quality or potentially very biased material. Papers were included if they provided specific data, policy or practice analyses, or substantial insight to key themes, regarding travel for necessary care. This approach was used to identify primary themes related to economic or legal considerations presented in available literature. Themes identified by the authors are part of a larger study examining medical travel in which various themes relating to medical travel were identified (2). Despite the existence of themes relating to economic and legal frameworks found in the literature, overall, we found that there had been no approach to synthesize this information to specifically inform a GHAP. We have, therefore, summarized the key considerations for establishing an economic and legal framework for GHAP, based on the themes identified. While taking into account that this is a non-exhaustive compilation of proposals, the current work provides the first step toward the development of an economic and legal framework for medical travel (such as GHAP).

### Economic Opportunities and Considerations

Medical travel may provide numerous financial incentives for health-care systems and travelers alike. In inbound countries, attracting medical travelers can serve as a mechanism to generate additional revenue (11). The resulting higher utilization of resources, such as hospitals and technical equipment, can contribute to cost reduction in health-care delivery (12). Furthermore, medical travel has the potential to contribute to economic growth through the creation of local job opportunities (13). A study in India investigated the effects of medical travel on the local population and highlighted benefits with regards to enhanced economic growth, revserse brain drain, and the increased investment in medical technologies and training (14). To further maximize the benefits for the local population, tax revenues accrued through linkages with tourism, insurance, food, and hotel business (12) could be allocated to the hospitals and health-care systems for domestic patients (15). Singapore and Cuba are useful examples, since the involvement of the respective countries in medical tourism, according to local authorities, has generated income benefiting the local population (16, 17). There is also evidence to suggest that due to the requirement to comply with international quality standards of care, there has been a trickle-down effect in inbound countries in terms of skills, technologies, and improved quality standards (1, 14). Finally, a liberalization of the market can exert competitive pressure on local health-care providers and consequently drive down costs by encouraging economies to maximize their comparative advantage (5).

Medical travel also has the capacity for negative financial consequences. A significant source of financial concern results from complications associated with medical treatment abroad (7). The potential spread of disease due to the unregulated movement of patients can create significant burden on national health systems and insurance companies (6). Another source of concern is that resources are primarily invested into private hospitals addressing the need of paying medical travelers, resulting in an inequitable two-tiered health system (12, 15, 18). For the local population, this can contribute to rising prices and limited access to health services (12, 15). This phenomenon is seen in Thailand, where a significant number of locals are unable to afford medical care in the private sector as a consequence of artificially high prices targeted toward medical travelers (19). Moreover, it may lead to understaffing of the local sector and increased strain on the public-care providers (14). Furthermore, increase in number of high-income patients traveling abroad for care may result in a decrease in revenue in the outbound country, as well as decline in political support for investment and development in local facilities and technology (20). Outbound countries may also have some obligation to subsidize medical travelers from their country, as outlined in the EU’s Patients’ Rights Directive (8).

### Key Elements for an Economic Framework

Based on the factors outlined in the previous section, Table 1 summarizes the economic risks, opportunities, and suggested key elements in the medical travel context. First, inbound countries must focus on limiting the rise of two-tiered systems. For example, a tax policy could be introduced to health-care providers, which would subsidize the local health-care system. Another solution could entail a flexible approach to pricing of medical procedures. Such interventions would support the local health infrastructure and limit the impact of market mechanisms, which otherwise poses disadvantages to local populations due to the increasing prices (21). Furthermore, establishing a solid economic framework could stimulate competition within inbound countries, which might subsequently result to lower domestic prices. In outbound countries, ensuring that medical travelers are reimbursed for the cost of treatment may increase access to medical care for patients (Verra et al., in review), as well as facilitate follow-up care in the traveler’s home country.

**Table 1:** **Economic opportunities and risks for medical travel and suggested framework elements**.

Inbound countries	
**Opportunities**	**Suggested framework elements**
1. Generate additional revenue for domestic health-care system	Tax policies to subsidize local health system
2. Create job opportunities	Flexible approach to pricing in inbound countries
3. Tax revenue sharing with domestic health-care system	Accrue tax revenues *via* hotel, food, etc.
**Risks**	**Suggested framework elements**
1. Spread of disease due to unregulated movement of patients	Improved quality standards
2. The development of an inequitable, two-tiered health system	See inbound opportunities 1 and 3

Outbound countries	

**Opportunities**	**Suggested framework elements**
1. Residents can seek out treatment that is costly/unavailable	Involvement/engagement in a GHAP
2. Increased availability of specialists	Facilitating more efficient division of labor and knowledge
**Risks**	**Suggested framework elements**
1. Decreased revenue as a result of patients traveling abroad	See outbound opportunities 1 and 2

### Legal Opportunities and Considerations

In order to protect patients and reduce the financial burden on health systems, regulatory oversight needs to be established for each step within the medical travel process. Regarding the early stages of the medical travel process (exploration and planning phases), legal and ethical considerations include providing information for patients, which now includes the booming market of third-party agencies, mediating between patients and prospective medical centers. Such organizations vary in terms of comprehensiveness of service but generally finalize travel arrangements, liaise with the clinics abroad, and make follow-up arrangements (22). As they often function as the first point of contact for patients, they play a significant role as information provider. Currently, there are no regulations in place to monitor the type and reliability of information provided by the third-party agencies, which has led to a market operating in a “regulatory vacuum” (23).

Several studies report shortcomings regarding the communication of medical information and records in the treatment and follow-up stages of travel (24, 25), as well as substandard follow-up care and social support (26, 27). Although the Patients’ Rights Directive, in theory, requires that all EU member states provide follow-up care (8), research shows that, in practice, physicians are frequently unaware of this requirement (28), and additional binding requirements to ensure continuity of care are not in place (9). There is also no consensus on international quality standards, which aim to reduce the differences in the quality level of treatment and hospital facilities (15, 20, 29, 30). As a consequence, there is a greater risk for the transmission of disease as well as the risk of complications pre- and postmedical treatment (9).

### Key Elements for a Legal Framework

With medical travelers moving across the world, it is imperative to establish a legal framework clearly outlining responsibilities and liabilities for each step of the medical travel process, especially with regards to transitions such as follow-up care and adverse events. Table 2 summarizes the legal risks, opportunities, and suggested key elements in the medical travel context. Given the broad nature of these considerations and the inclusion of patient safety and well-being considerations, we note that these include some ethical elements but have given them the general term “legal” considerations. Of particular importance throughout the entire medical travel process should be the definition and implementation of internationally standardized minimum quality standards (30). This process should encompass an external evaluation and associated accreditation program to ensure that health-care facilities comply with minimum standards (23). In order to avoid the emergence of various accrediting bodies and discrepancies in the accreditation process, the establishment of an international authority would be necessary to oversee the process (for instance, the International Society for Quality in Healthcare) (31). Due to the rapidly developing market of third-party agencies, there is an urgent call for an establishment and maintenance of quality standards in the domain of third-party health-care providers (30).

**Table 2:** **Legal opportunities and risks for medical travel and suggested framework elements**.

Inbound countries	
**Opportunities**	**Suggested framework elements**
1. Adherence to international quality standards	Involvement/engagement in a GHAP
2. Provision of social support during treatment process	
**Risks**	**Suggested framework elements**
1. Incorrect communication of medical information	Guidelines regarding handling of patient data
2. Discrepancies in international quality standards	Establishment of an authority to oversee standards process

Outbound countries	

**Opportunities**	**Suggested framework elements**
1. Standardization of services provided by third-party agencies	Involvement/engagement in a GHAP
2. Continuity of care	Implementation of guidelines that ensure follow-up care
**Risks**	**Suggested framework elements**
1. Financial implications of adverse events and unregulated developments	Outlined responsibilities for each step of medical travel

In order to facilitate the treatment process and prevent complications, the communication of medical information is a key element to consider in the development of a legal framework. A failure to transmit medical notes to the treating center abroad could be avoided by the use of a common online platform for health-care staff and patients. This platform could be utilized for medical communication as well as transfer of medical records (32). Regulations should outline requirements in terms of handling of sensitive patient data and risk communication (33).

Additionally, legal frameworks focused on the provision of social support need to be considered both during the treatment and follow-up processes. Social support can decrease stress, improve patients’ well-being while traveling, and improve the recovery process (34, 35). With this in mind, the definition of juridical facilitation of travel arrangements could be of great benefit to medical travelers. This could entail the provision of subsidies for costs of an accompanying significant other, as well as less stringent visa procedures, when accompanying a relative during the medical travel process.

## Concluding Remarks

The implementation of a GHAP is crucial to ensure safe and economically beneficial medical travel for a broader population. In inbound countries, the promotion of medical travel has the capacity to counter increasing costs of health care by creating new markets and increased revenue for health services, and lead to more efficient use of resources and labor benefiting local populations, economies, and health-care systems. In outbound countries, medical travel can provide access to treatment that is more affordable or unavailable domestically. Further, legal consequences must be considered at each phase of the medical travel process. Legal considerations include the standardization of visa procedures, the implementation of international quality standards, the accreditation of health-care providers and third-party agencies, the protection of the local population, and the monitoring of information provided to patients to ensure informed decision making for the patients and the families involved. Fairness, equal access to treatments for medical travelers and local populations, equity, and long-term sustainability are key components when developing a GHAP framework. Considering the existing risk for all stakeholders, it is essential that existing economic and legal barriers to medical travel are accounted for in the establishment of a comprehensive GHAP.

## Author Contributions

All authors contributed to the data collection for the paper. NR, JT, SB, OK, KR, and SHK contributed to the writing of the paper. In addition, KR, CD, and SHK contributed to the design of the study.

## Conflict of Interest Statement

The authors declare that the research was conducted in the absence of any commercial or financial relationships that could be construed as a potential conflict of interest.
